# Supplementary material for: Estimating an EQ-5D-3L Value Set for Romania Using Time Trade-Off
Source: Int J Environ Res Public Health. 2021 Jul 11;18(14):7415. doi: 10.3390/ijerph18147415 (PMC8306594; doi:10.3390/ijerph18147415)
Supplement: Supplementary file 1 [file ijerph-18-07415-s001.zip › ijerph-1255534-SI.pdf]

Table S1. Respondents' characteristics – categorical variables, dataset V1

Variable	Category	V1 (n=1649)		Weighted V1 (n=1649)		General population
		n	%	n	%	%
MO	No problems	1371	83.1	1333	80.83	
	Some problems	374	16.62	302	18.88	N/A
	Confined to bed	4	0.24	5	0.28	
SC	No problems	1512	91.69	1474	89.40	N/A
	Some problems	133	8.07	170	10.32	
	Unable	4	0.24	5	0.28	
UA	No problems	1396	84.66	1348	81.74	
	Some problems	242	14.68	288	17.44	N/A
	Unable	11	0.67	13	0.82	
PD	No problems	1206	73.14	1179	71.50	
	Some problems	438	26.56	464	28.12	N/A
	Extreme problems	5	0.30	6	0.38	
AD	No problems	1360	82.47	1347	81.71	
	Some problems	241	14.61	257	15.58	N/A
	Extreme problems	48	2.91	45	2.71	
Gender	Female	1072	65.01	857	51.99	52%
Residence area	Urban	1212	73.50	895	54.28	55.20%
Education level	No formal education	7	0.42	13	0.82	2%
	Low	192	11.64	255	15.46	36.90%
	Medium	830	50.33	877	53.21	45.20%
	Tertiary	611	37.05	494	29.95	15.90%
	No response	9	0.55	9	0.56	
Occupation	Employed	973	59.01	867	52.55	52.10%
	Unemployed	35	2.12	54	3.29	3.90%
	Retired	415	25.17	442	26.79	26.20%
	Stay at home/domestic	117	7.10	147	8.91	7.10%
	In education	92	5.58	111	6.72	4.80%
	No response	17	1.03	28	1.73	
Income	Below the average	705	42.75	773	46.86	41.39%
	Average	298	18.07	278	16.83	30.75%
	Above the average	513	31.11	447	27.10	27.86%
	No response	133	8.07	152	9.21	

V1 Dataset contains all valid responses and corresponds to exclusion criterion (interviews of suspect quality performed by interviewers that were excluded from the team of interviewers due to non-compliance).

Table S2. All models that were tested.

Model	Consistency of parameters achieved	Non-Significant parameters
OLS	Yes	MO2
ROLS	Yes	0
RME	Yes	MO2

IME	Yes	MO2; constant
TOB	Yes	MO2
TOBRME	Yes	MO2
IRM	Yes	0
IRMC	Yes	0
IRMCRME	Yes	M02
IRMCIME	Yes	MO2; constant
RCM	Yes	MO2

OLS, ordinary least-squares; ROLS, robust ordinary least-squares; RME, respondent-level mixed effects; IME, interviewer-level mixed effects; TOB, tobit; TOBRME, tobit with respondent-level mixed effects; IRM, interval regression model; IRMC, interval regression model censored at -1; IRMCRME, interval regression model censored at -1 with respondent-level mixed effects; IRMCIME, interval regression model censored at -1 with interviewer-level mixed effects; RCM, random coefficient model.

Table S3. Full value set model for the Romanian version of EQ-5D-3L.

Independent variables of the model	Coefficient	SE	p-value	95% CI	
(Constant)	0.032	0.005	0.000	0.022	0.042
MO2	0.038	0.005	0.000	0.027	0.048
MO3	0.394	0.013	0.000	0.369	0.420
SC2	0.040	0.006	0.000	0.029	0.052
SC3	0.206	0.010	0.000	0.187	0.225
UA2	0.044	0.005	0.000	0.034	0.055
UA3	0.189	0.011	0.000	0.167	0.212
PD2	0.072	0.006	0.000	0.060	0.083
PD3	0.371	0.012	0.000	0.348	0.394
AD2	0.054	0.006	0.000	0.042	0.065
AD3	0.206	0.010	0.000	0.187	0.224
**Model for lnsigma**					
(Constant)	-2.256	0.028	0.000	-2.312	-2.201
MO2	-0.060	0.025	0.019	-0.109	-0.010
MO3	0.560	0.027	0.000	0.507	0.613
SC2	0.045	0.027	0.091	-0.007	0.098
SC3	0.416	0.028	0.000	0.361	0.472
UA2	0.009	0.027	0.730	-0.044	0.062
UA3	0.357	0.027	0.000	0.304	0.410
PD2	0.148	0.027	0.000	0.095	0.201
PD3	0.591	0.027	0.000	0.538	0.643
AD2	0.144	0.026	0.000	0.092	0.195
AD3	0.599	0.027	0.000	0.548	0.651

Table S4. Predicted values for the EQ-5D-3L – Romanian model (RO model)

State	RO model	State	RO model	State	RO model	State	RO model
11111	1.000	12211	0.883	13311	0.572	22111	0.890
11112	0.914	12212	0.829	13312	0.518	22112	0.836
11113	0.762	12213	0.677	13313	0.367	22113	0.684
11121	0.896	12221	0.811	13321	0.501	22121	0.818
11122	0.842	12222	0.757	13322	0.447	22122	0.764
11123	0.691	12223	0.606	13323	0.295	22123	0.612
11131	0.596	12231	0.512	13331	0.201	22131	0.518
11132	0.542	12232	0.458	13332	0.147	22132	0.464
11133	0.391	12233	0.306	13333	-0.005	22133	0.313
11211	0.923	12311	0.738	21111	0.930	22211	0.845
11212	0.870	12312	0.684	21112	0.876	22212	0.791
11213	0.718	12313	0.532	21113	0.724	22213	0.640
11221	0.852	12321	0.666	21121	0.858	22221	0.774
11222	0.798	12322	0.612	21122	0.804	22222	0.720
11223	0.646	12323	0.461	21123	0.653	22223	0.568
11231	0.552	12331	0.366	21131	0.559	22231	0.474
11232	0.498	12332	0.312	21132	0.505	22232	0.420
11233	0.347	12333	0.161	21133	0.353	22233	0.268
11311	0.778	13111	0.762	21211	0.886	22311	0.700
11312	0.724	13112	0.708	21212	0.832	22312	0.646
11313	0.573	13113	0.556	21213	0.680	22313	0.495
11321	0.707	13121	0.690	21221	0.814	22321	0.628
11322	0.653	13122	0.636	21222	0.760	22322	0.574
11323	0.501	13123	0.484	21223	0.609	22323	0.423
11331	0.407	13131	0.390	21231	0.514	22331	0.329
11332	0.353	13132	0.336	21232	0.461	22332	0.275
11333	0.201	13133	0.185	21233	0.309	22333	0.123
12111	0.927	13211	0.717	21311	0.741	23111	0.724
12112	0.873	13212	0.664	21312	0.687	23112	0.670
12113	0.722	13213	0.512	21313	0.535	23113	0.518
12121	0.856	13221	0.646	21321	0.669	23121	0.652
12122	0.802	13222	0.592	21322	0.615	23122	0.598
12123	0.650	13223	0.440	21323	0.463	23123	0.447
12131	0.556	13231	0.346	21331	0.369	23131	0.353
12132	0.502	13232	0.292	21332	0.315	23132	0.299
12133	0.350	13233	0.141	21333	0.164	23133	0.147

Predicted values for the EQ-5D-3L – Romanian model (continued)

State	RO model	State	RO model	State	RO model
23211	0.680	31311	0.384	33111	0.367
23212	0.626	31312	0.330	33112	0.314
23213	0.474	31313	0.179	33113	0.162
23221	0.608	31321	0.312	33121	0.296
23222	0.554	31322	0.258	33122	0.242
23223	0.403	31323	0.107	33123	0.090
23231	0.308	31331	0.013	33131	-0.004
23232	0.254	31332	-0.041	33132	-0.058
23233	0.103	31333	-0.193	33133	-0.209
23311	0.534	32111	0.533	33211	0.323
23312	0.481	32112	0.479	33212	0.269
23313	0.329	32113	0.328	33213	0.118
23321	0.463	32121	0.461	33221	0.252
23322	0.409	32122	0.407	33222	0.198
23323	0.257	32123	0.256	33223	0.046
23331	0.163	32131	0.162	33231	-0.048
23332	0.109	32132	0.108	33232	-0.102
23333	-0.042	32133	-0.044	33233	-0.254
31111	0.574	32211	0.489	33311	0.178
31112	0.520	32212	0.435	33312	0.124
31113	0.368	32213	0.283	33313	-0.028
31121	0.502	32221	0.417	33321	0.106
31122	0.448	32222	0.363	33322	0.052
31123	0.296	32223	0.212	33323	-0.099
31131	0.202	32231	0.118	33331	-0.193
31132	0.148	32232	0.064	33332	-0.247
31133	-0.003	32233	-0.088	33333	-0.399
31211	0.529	32311	0.344		
31212	0.475	32312	0.290		
31213	0.324	32313	0.138		
31221	0.458	32321	0.272		
31222	0.404	32322	0.218		
31223	0.252	32323	0.066		
31231	0.158	32331	-0.028		
31232	0.104	32332	-0.082		
31233	-0.048	32333	-0.233		

Table S5. Full RO model run on dataset V1

Independent variables of the model	Coefficient	SE	p-value	95% CI	
**(Constant)**	0.032	0.005	0.000	0.022	0.043
MO2	0.037	0.006	0.000	0.026	0.048
MO3	0.389	0.013	0.000	0.364	0.414
SC2	0.044	0.006	0.000	0.032	0.056
SC3	0.207	0.010	0.000	0.187	0.226
UA2	0.043	0.005	0.000	0.032	0.054
UA3	0.182	0.011	0.000	0.160	0.205
PD2	0.069	0.006	0.000	0.057	0.080
PD3	0.355	0.012	0.000	0.332	0.377
AD2	0.054	0.006	0.000	0.042	0.065
AD3	0.209	0.010	0.000	0.190	0.227
**Model for lnsigma**					
(Constant)	-2.208	0.027	0.000	-2.262	-2.155
MO2	-0.046	0.025	0.061	-0.095	0.002
MO3	0.535	0.026	0.000	0.484	0.587
SC2	0.106	0.026	0.000	0.055	0.156
SC3	0.428	0.028	0.000	0.374	0.482
UA2	-0.020	0.026	0.437	-0.072	0.031
UA3	0.343	0.026	0.000	0.292	0.394
PD2	0.147	0.026	0.000	0.096	0.199
PD3	0.577	0.026	0.000	0.526	0.629
AD2	0.108	0.026	0.000	0.058	0.159
AD3	0.560	0.026	0.000	0.509	0.610

Table S6. Full RO model run on weighted V3 dataset

Independent variables of the model	Coefficient	SE	p-value	95% CI	
(Constant)	0.039	0.006	0.000	0.027	0.050
MO2	0.031	0.007	0.000	0.018	0.045
MO3	0.416	0.019	0.000	0.379	0.453
SC2	0.046	0.007	0.000	0.033	0.059
SC3	0.228	0.017	0.000	0.196	0.261
UA2	0.041	0.006	0.000	0.028	0.053
UA3	0.183	0.015	0.000	0.154	0.212
PD2	0.070	0.007	0.000	0.057	0.083
PD3	0.359	0.016	0.000	0.328	0.390
AD2	0.051	0.007	0.000	0.037	0.065
AD3	0.200	0.012	0.000	0.177	0.224
**Model for lnsigma**					
(Constant)	-2.242	0.063	0.000	-2.365	-2.120
MO2	-0.073	0.073	0.320	-0.216	0.071
MO3	0.568	0.064	0.000	0.443	0.694
SC2	0.094	0.049	0.054	-0.002	0.190
SC3	0.506	0.098	0.000	0.314	0.699
UA2	0.045	0.065	0.491	-0.083	0.173
UA3	0.338	0.061	0.000	0.217	0.458
PD2	0.143	0.068	0.036	0.009	0.277
PD3	0.591	0.058	0.000	0.477	0.705
AD2	0.063	0.073	0.392	-0.081	0.206
AD3	0.529	0.056	0.000	0.418	0.639
